# Diagnostic and molecular testing patterns in patients with newly diagnosed acute myeloid leukemia in the Connect^®^ MDS/AML Disease Registry

**DOI:** 10.1002/jha2.16

**Published:** 2020-06-30

**Authors:** Daniel A. Pollyea, Tracy I. George, Mehrdad Abedi, Rafael Bejar, Christopher R. Cogle, Kathryn Foucar, Guillermo Garcia‐Manero, David L. Grinblatt, Rami S. Komrokji, Jaroslaw P. Maciejewski, Dennis A. Revicki, Gail J. Roboz, Michael R. Savona, Bart L. Scott, Mikkael A. Sekeres, Michael A. Thompson, Sandra E. Kurtin, Chrystal U. Louis, Melissa Nifenecker, E. Dawn Flick, Arlene S. Swern, Pavel Kiselev, David P. Steensma, Harry P. Erba

**Affiliations:** ^1^ Department of Medicine Division of Hematology University of Colorado Aurora Colorado USA; ^2^ University of Utah and ARUP Laboratories Salt Lake City Utah USA; ^3^ University of California Davis Sacramento California USA; ^4^ Moores Cancer Center University of California San Diego Health La Jolla California USA; ^5^ University of Florida Gainesville Florida USA; ^6^ University of New Mexico Health Sciences Center Albuquerque New Mexico USA; ^7^ MD Anderson Cancer Center University of Texas Houston Texas USA; ^8^ NorthShore University Health System Evanston Illinois USA; ^9^ H. Lee Moffitt Cancer Center Tampa Florida USA; ^10^ Cleveland Clinic Foundation Cleveland Ohio USA; ^11^ Evidera Bethesda Maryland USA; ^12^ Weill Cornell College of Medicine New York New York USA; ^13^ Vanderbilt‐Ingram Cancer Center Vanderbilt University School of Medicine Nashville Tennessee USA; ^14^ Fred Hutchinson Cancer Research Center Seattle Washington USA; ^15^ Advocate Aurora Health Advocate Aurora Research Institute Milwaukee Wisconsin USA; ^16^ University of Arizona Cancer Center Tucson Arizona USA; ^17^ Bristol‐Myers Squibb Summit New Jersey USA; ^18^ Bristol‐Myers Squibb San Francisco California USA; ^19^ Dana‐Farber Cancer Institute Boston Massachusetts USA; ^20^ Duke University Durham North Carolina USA

**Keywords:** acute myeloid leukemia, diagnostic testing, leukemia diagnosis, leukemia therapy, molecular testing, registry

## Abstract

Diagnostic and molecular genetic testing are key in advancing the treatment of acute myeloid leukemia (AML), yet little is known about testing patterns outside of clinical trials, especially in older patients. We analyzed diagnostic and molecular testing patterns over time in 565 patients aged ≥ 55 years with newly diagnosed AML enrolled in the Connect^®^ MDS/AML Disease Registry (NCT01688011) in the United States. Diagnostic data were recorded at enrolment and compared with published guidelines. The percentage of bone marrow blasts was reported for 82.1% of patients, and cellularity was the most commonly reported bone marrow morphological feature. Flow cytometry, karyotyping, molecular testing, and fluorescence in situ hybridization were performed in 98.8%, 95.4%, 75.9%, and 75.7% of patients, respectively. Molecular testing was done more frequently at academic than community/government sites (84.3% vs 70.2%; *P *< .001). Enrolment to the Registry after 2016 was significantly associated with molecular testing at academic sites (odds ratio [OR] 2.59; *P* = .023) and at community/government sites (OR 4.85; *P *< .001) in logistic regression analyses. Better understanding of practice patterns may identify unmet needs and inform institutional protocols regarding the diagnosis of patients with AML.

## 1 INTRODUCTION

Acute myeloid leukemia (AML) is the most common acute leukemia in adults [1] occurring in 4.3 in 100 000 individuals in the United States [2]. AML is a heterogeneous disease with numerous cytogenetic and molecular features influencing prognosis [3, 4, 5].

According to current guidelines, accurate diagnosis and initial evaluation of AML requires clinical, morphological, cytogenetic, and molecular genetic assessment [3, 4, 5, 6]. Approximately 50% of patients with AML have an abnormal karyotype, and a median of 3 gene mutations per patient [7]. More mutations are generally associated with worse prognosis; however, prognosis depends heavily on which genes are mutated [8]. The addition of diagnostic molecular genetic testing results to risk stratification models has enhanced prognostication [8, 9] and, with the advent of targeted therapies, has become essential in making treatment decisions. Since 2017, four targeted therapies have been approved for treatment of AML: the *FLT3* inhibitors midostaurin [10, 11] and gilteritinib [12] for newly diagnosed and relapsed/refractory patients, respectively, the *IDH1* inhibitor ivosidenib [13, 14, 15] for newly diagnosed and relapsed/refractory patients, and the *IDH2* inhibitor enasidenib [16] for relapsed/refractory patients. Enasidenib was evaluated in patients with newly diagnosed *IDH2*+ AML [17], and sorafenib and dasatinib are used off‐label for patients with *FLT3‐ITD* and *KIT* mutations, respectively [12, 18]. Quizartinib and crenolanib are *FLT3* inhibitors under clinical investigation [19, 20], as is FT‐2102 for *IDH1* [21] and APR‐246 for *TP53* [22].

Despite the critical roles that diagnostic and molecular genetic testing play in AML treatment [23], our understanding of how these tests are used, particularly in older patients who are often excluded from clinical trials, is limited. Outcomes in older patients with AML remain poor; median survival ranges from 2.5 to 24.5 months, depending on risk group [24, 25]. Several factors contribute to poor outcomes, including an increased probability of drug resistance and unfavorable cytogenetics [3]. Older patients often receive less intensive therapy due to perceived frailty, which can lead to worse outcomes [25, 26].

We summarize diagnostic and molecular testing patterns by site, enrolment year, and adherence to guidelines in patients with AML enrolled in the Connect^®^ MDS/AML Disease Registry.

## 2 MATERIALS AND METHODS

### 2.1 Study design and setting

The Connect^®^ MDS/AML Disease Registry is a large, ongoing, US, multicenter, prospective observational cohort study of patients with newly diagnosed AML, MDS, or idiopathic cytopenia of undetermined significance (NCT01688011). A full description of the study design and objectives was reported previously [27]. The study is non‐interventional; all medical care is performed at the discretion of the treating clinicians in accordance with standard clinical practice at each site. The Registry was approved by a central institutional review board (IRB; Advarra Review IRB, Seattle, WA, USA) or the IRB at each site. All patients provided written informed consent. Enrolment began in December 2013 and will continue until approximately 2100 patients have enrolled, including 700 patients with AML, from approximately 150 to 200 sites.

### 2.2 Participants

To minimize selection bias, consecutive patients with AML seen at participating sites are invited to enrol in the Registry. Patients aged ≥55 years and newly diagnosed with primary or secondary AML, as per the 2008 revised World Health Organization (WHO) guidelines [28], within 60 days of enrolment are eligible. Local diagnosis is confirmed by independent central pathology review of laboratory reports. Bone marrow (BM) aspirate and biopsy results are not required if laboratory results indicate ≥20% myeloblasts in the peripheral blood. Patients are excluded if they have suspected or diagnosed acute promyelocytic leukemia (APL). Patients with AML are also excluded if they have received active, disease‐modifying treatment for ≥14 days prior to enrolment. Patients receiving only supportive care may enroll.

### 2.3 Measurements

Patient data are collected in an electronic data capture system at baseline and every 3 months for up to 8 years, or until early study termination, patient withdrawal, or death. Data include patient characteristics, comorbidities (assessed by the Adult Comorbidity Evaluation form ACE‐27 [29], frailty evaluation (assessed by the Canadian Study of Health and Aging [CSHA] Clinical Frailty Scale [30, 31]), diagnostic testing results, treatment, and outcomes. Molecular genetic testing data presented are based on the 19 genes (Figure S1) included in the study electronic database.

### 2.4 Statistical analyses

Demographics and clinical characteristics were summarized using descriptive statistics. Categorical values were reported as proportions of the total Registry cohort. *P‐*values were calculated for pairwise comparisons of baseline variables at academic and community/government sites using chi‐square test, Fisher exact test (for categorical values) and Wilcoxon test (for median values). Factors associated with molecular genetic testing of 19 specific mutations were evaluated using univariate and multivariable logistic regression analysis with variable selection based on chi‐square statistics. Variables significant at *P *< .1 in univariate analyses were included in multivariable analyses. All statistical analyses were conducted using SAS^®^ version 9.2 or higher (SAS Institute, Cary, NC, USA). Statistical testing was conducted at an α = .05 (two‐sided) significance level. The data cut‐off for this analysis was 8 March 2019.

## 3 RESULTS

### 3.1 Baseline characteristics

Baseline characteristics and testing data collected from patients enrolled in the Registry from 12 December 2013 to the data cut‐off (8 March 2019) were included in this analysis. In total, 565 patients with newly diagnosed AML were enrolled in the Registry across 116 sites in the US (Table 1). Median age was 70 years (range 55–92 years) and 72.7% were aged ≥65 years. A total of 229 patients (40.5%) were enrolled from academic sites and 336 (59.5%) from community/government sites. Academic centers in the Northeast and South enrolled most patients, and community/government sites more commonly enrolled patients in the Midwest and South. Most patients had an ECOG PS of 0‐1 (72.5%) and a mild (1‐4) CSHA Clinical Frailty Scale score (84.2%). About 40% of patients had an ACE‐27 comorbidity score of 2‐3.

**TABLE 1:** Baseline characteristics

Characteristic	All patients with AML (N = 565)	Academic sites (n = 229)	Community/ government sites (n = 336)	*P* value^*^
Age,^†^ years	N = 565	n = 229	n = 336	
Median (range)	70 (55–92)	69 (55–87)	72 (55–92)	.0001^‡^
Age ≥ 65 years, n (%)	411 (72.7)	153 (66.8)	258 (76.8)	.009
Sex	N = 565	n = 229	n = 336	
Male, n (%)	354 (62.7)	148 (64.6)	206 (61.3)	NS
Race, n (%)^§^	N = 564	n = 229	n = 335	
White	473 (83.9)	191 (83.4)	282 (84.2)	NS
Black	37 (6.6)	19 (8.3)	18 (5.4)	
Other	10 (1.8)	5 (2.2)	5 (1.5)	
Not specified	45 (8.0)	14 (6.1)	31 (9.3)	
US geographic region, n (%)	n = 565	n = 229	n = 336	<.0001
South	235 (41.6)	112 (48.9)	123 (36.6)	
Midwest	138 (24.4)	42 (18.3)	96 (28.6)	
West	99 (17.5)	17 (7.4)	82 (24.4)	
Northeast	93 (16.5)	58 (25.3)	35 (10.4)	
Primary insurance, n (%)	n = 551	n = 221	n = 330	.0144^‖^
Medicare	323 (58.6)	116 (52.5)	207 (62.7)	
Medicaid	12 (2.2)	9 (4.1)	3 (0.9)	
Private HMO/PPO	118 (21.4)	58 (26.2)	60 (18.2)	
Private other	52 (9.4)	20 (9.0)	32 (9.7)	
Veterans/military	12 (2.2)	2 (0.9)	10 (3.0)	
Other	32 (5.8)	15 (6.8)	17 (5.2)	
Unknown	2 (0.4)	1 (0.5)	1 (0.3)	
Time from diagnosis to enrolment, days	N = 565	n = 229	n = 336	
Median (range)	7 (‐3–64)	6 (‐1–52)	10 (‐3–64)	<.0001^‡^
ECOG PS, n (%)	n = 483	n = 182	n = 301	
0–1	350 (72.5)	127 (69.8)	223 (74.1)	NS
≥ 2	133 (27.5)	55 (30.2)	78 (25.9)	
CSHA Clinical Frailty Scale score, n (%)	n = 360	n = 120	n = 240	
Mild (1–4)	303 (84.2)	101 (84.2)	202 (84.2)	NS
Moderate (5–6)	47 (13.1)	14 (11.7)	33 (13.8)	
Severe (7–9)	10 (2.8)	5 (4.2)	5 (2.1)	
Comorbidity score,^¶^ n (%)	n = 466	n = 186	n = 280	NS
0–1	278 (59.7)	122 (65.6)	156 (55.7)	
2–3	188 (40.3)	64 (34.4)	124 (44.3)	

Rounding of numbers may cause totals to be < or > 100%.

Abbreviations: AML, acute myeloid leukemia; CSHA, Canadian Study of Health and Ageing; ECOG PS, Eastern Cooperative Oncology Group performance status; HMO, health maintenance organization; NS, not significant; PPO, preferred provider organization.

* Academic versus community/government sites, chi‐square test, unless otherwise stated.

† Patients ≥ 55 years eligible for enrolment.

‡ Median two‐sample test, two‐sided Pr > z.

§ White versus Black versus other (American Indian/Asian/Pacific Islander/Other) versus Not specified.

‖ Private insurance versus Medicare versus all other types combined.

¶ Comorbidities assessed based on Adult Comorbidity Evaluation‐27 (ACE‐27).

### 3.2 Bone marrow blast assessment and morphology

**FIGURE 1:**
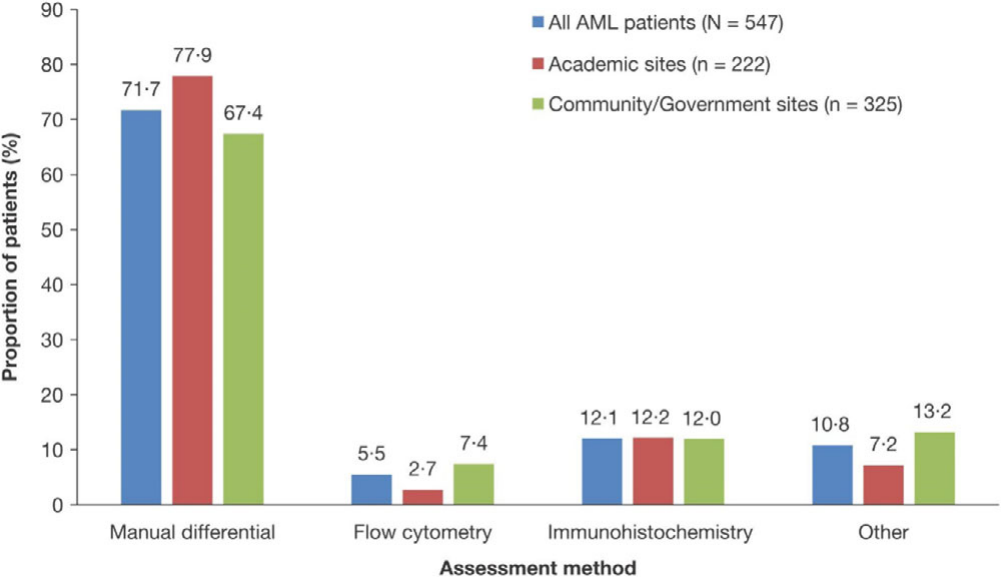
Bone marrow blast assessment. "Other" refers to estimates of bone marrow blast percentage made from aspirate smear or biopsy sections. AML, acute myeloid leukemia

BM blast percentage was reported for 82.1% of patients. BM blasts were measured by a manual count from the aspirate or touch preparation in 71.7% of patients (Figure 1), as recommended by the WHO guidelines [28, 32]. Cellularity was the most commonly reported BM morphological feature, assessed in 93.6% of patients. Dysplasia was reported in 42.4‐62.9% of patients, fibrosis in 44.2% of patients, and ring sideroblasts in 52.4% of patients (Table 2).

**TABLE 2:** Proportion of patients with reports on bone marrow morphology

Test, n (%)		All patients with AML (N = 561)	Academic sites (n = 227)	Community/ government sites (n = 334)	*P* value^*^
Erythroid dysplasia	Reported	326 (58.1)	133 (58.6)	193 (57.8)	0.8494
	Not reported	235 (41.9)	94 (41.4)	141 (42.2)	
Megakaryocytic dysplasia	Reported	353 (62.9)	153 (67.4)	200 (59.9)	0.0703
	Not reported	208 (37.1)	74 (32.6)	134 (40.1)	
Neutrophil dysplasia	Reported	238 (42.4)	97 (42.7)	141 (42.2)	0.9034
	Not reported	323 (57.6)	130 (57.3)	193 (57.8)	
Auer rods	Reported	232 (41.4)	84 (37.0)	148 (44.3)	0.0845
	Not reported	329 (58.6)	143 (63.0)	186 (55.7)	
Ring sideroblasts^†^	Reported	294 (52.4)	111 (48.9)	183 (54.8)	0.1284
	Not reported	256 (45.6)	113 (49.8)	143 (42.8)	
Cellularity	Reported	525 (93.6)	213 (93.8)	312 (93.4)	0.8423
	Not reported	36 (6.4)	14 (6.2)	22 (6.6)	
Fibrosis	Reported	248 (44.2)	106 (46.7)	142 (42.5)	0.3277
	Not reported	313 (55.8)	121 (53.3)	192 (57.5)	

For 4 patients (2 academic sites and 2 community/government sites) responses were not provided.

AML, acute myeloid leukaemia.

* Academic versus community/government sites.

† For 11 patients (3 academic sites, 8 community/government sites), ring sideroblast testing was considered not applicable.

### 3.3 Ancillary testing, including molecular genetic testing

**FIGURE 2:**
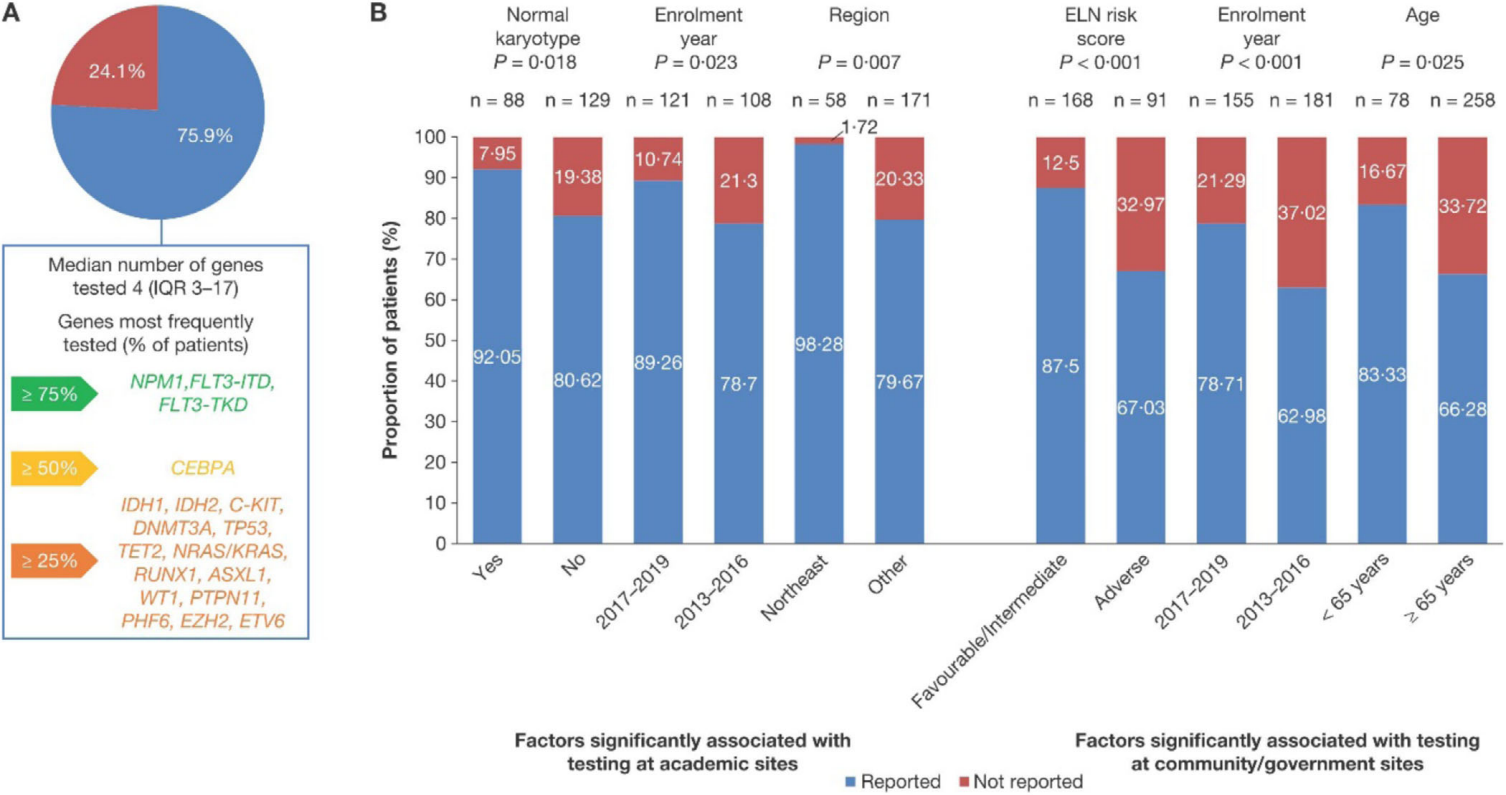
Differences in molecular genetic testing rates in Registry patients with newly diagnosed AML. (A) Proportion of patients with AML who received molecular testing, and most frequently tested genes from the 19 that are included in the study electronic database. (B) Factors significantly associated with molecular testing at academic and community/government sites, as determined by multivariable testing. Other refers to South, Midwestern and West US regions. AML, acute myeloid leukaemia; ELN, European Leukemia Network

Conventional karyotyping, flow cytometry, and fluorescence in situ hybridization (FISH) were reported in 95.4%, 98.8%, and 75.7% of patients, respectively. Molecular genetic testing was reported in 429 patients (75.9%) (Table 3) and was done more frequently at academic than community/government sites (84.3% vs 70.2%; *P *= .0001). Only 3.2% of patients underwent FISH or molecular genetic testing without conventional karyotyping. Of 429 patients who underwent molecular testing, blood specimens were analyzed for 89 patients and BM for 346 patients (Table 4). Median time from sample collection to reporting of molecular testing results for both blood and BM samples was 7 days. The time from BM sample collection to reporting of the results was longer for patients enrolled later in the Registry (2017‐2019), versus those enrolled earlier (2013‐2016; 8.5 vs 7 days, respectively; *P *= .007). For blood samples no significant difference in turnaround time was observed. There was variability in the number of genes tested (median 4, interquartile range [IQR: 3–17] from a total of 19 genes listed in Figure S1; Figure 2A). The most frequently tested mutations were *FLT3‐ITD* (87.9%), *NPM1* (79.7%), *FLT3‐TKD* (77.9%), and *CEBPA* (61.1%), consistent with National Comprehensive Cancer Network^©^ (NCCN) molecular testing recommendations [33] (Figure S1).

**TABLE 3:** Ancillary testing by academic versus community/government sites

n (%)	All patients with AML (N = 565)	Academic sites (n = 229)	Community/ government sites (n = 336)	*P* value^*^
Conventional karyotype testing	N = 565	n = 229	n = 336	
Yes	539 (95.4)	220 (96.1)	319 (94.9)	NS
No	26 (4.6)	9 (3.9)	17 (5.1)	
Flow cytometry performed	N = 565	n = 229	n = 336	
Yes	558 (98.8)	225 (98.3)	333 (99.1)	NS
No	7 (1.2)	4 (1.7)	3 (0.9)	
FISH analysis	n = 564	n = 229	n = 335	
Yes	427 (75.7)	171 (74.7)	256 (76.4)	NS
No	137 (24.3)	58 (25.3)	79 (23.6)	
Molecular genetic testing	N = 565	n = 229	n = 336	
Yes	429 (75.9)	193 (84.3)	236 (70.2)	0.0001
No	136 (24.1)	36 (15.7)	100 (29.8)	

AML, acute myeloid leukaemia; FISH, fluorescence in situ hybridisation; NS, not significant.

* Academic versus community/government sites.

**TABLE 4:** Summary of molecular genetic testing

	**Patients who received molecular testing (N = 429)**	
	**Blood specimen (n = 89)**	**Bone marrow specimen (n = 345)** ^*^
Median time from date of specimen collection to date of report, days (IQR)	7.0 (4.0–11.0)	7.0 (5.0–12.0)
Type of testing, n (%)	**n = 89**	**n = 346**
PCR‐based	61 (68.5)	221 (63.9)
Sanger sequencing	9 (10.1)	41 (11.8)
Next‐generation sequencing	30 (33.7)	158 (45.7)
Other	4 (4.5)	6 (1.7)

IQR, interquartile range.

* A total of 346 patients received molecular testing on bone marrow specimens; dates of reporting were missing for 1 patient.

At academic sites, univariate analysis identified five factors associated with molecular genetic testing: enrolment year, geographic region, age, normal karyotype, and European Leukemia Network (ELN) risk score. In multivariable analysis, factors significantly associated with higher testing rates were residing in the Northeast versus "Other" regions (odds ratio [OR] 16.13; 95% confidence interval [CI] 2.11 to 125.00; *P* = .007), normal karyotype (OR 3.01; 95% CI 1.20‐7.52; *P* = .018), and enrolment in 2017‐2019 versus earlier enrolment (OR 2.59; 95% CI 1.14‐5.88; *P* = .023) (Figure 2B).

At community/government sites, univariate analysis identified seven factors associated with molecular genetic testing: enrolment year, region, age, insurance type, normal karyotype, ELN risk score, and comorbidity score. In multivariable analysis, factors significantly associated with higher testing rates included enrolment in 2017‐2019 versus earlier enrolment (OR 4.85; 95% CI 2.25‐10.53; *P* < .001), a favorable or intermediate ELN risk score vs adverse risk score (OR 3.45; 95% CI 1.77‐6.71; *P* < .001), and age < 65 years vs ≥65 years (OR 2.80; 95% CI 1.138‐6.891; *P* = .025). The only overlapping independent predictor of molecular testing at both academic and community/government sites was enrolment year (Figure 2A).

### 3.4 Conformity with molecular genetic tests recommended in the ASH/CAP guidelines

**FIGURE 3:**
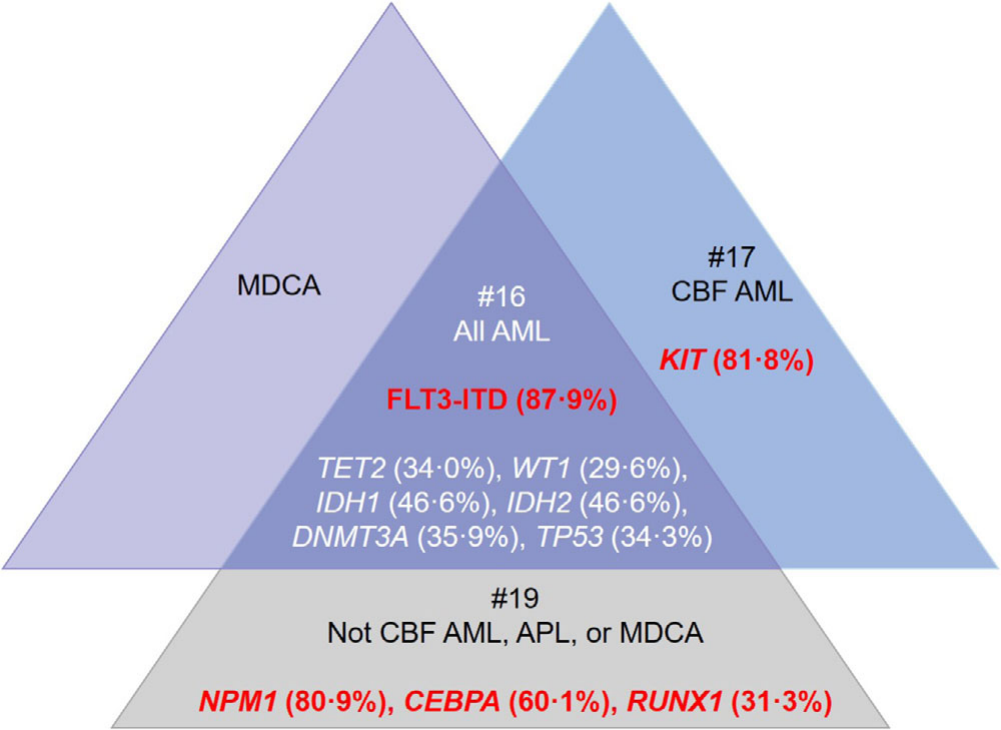
Frequency of ASH/CAP guideline‐recommended tests among patients who received any molecular genetic testing (n = 429). Recommendation #16 of the ASH/CAP guidelines recommends that all patients with AML should be tested for *FLT3*. As per recommendation #17, those with CBF‐AML should also be assessed for *KIT* mutations. Recommendation #19 notes that patients who do not have CBF‐AML, APL, or AML associated with MDCA should undergo mutational analysis for *NPM1*, *CEBPA*, and *RUNX1*. Text listed in red refers to the proportion of patients who received testing that is strongly recommended in the ASH/CAP guidelines, while white text refers to the proportion of patients who received testing that is recommended. AML, acute myeloid leukaemia; ASH/CAP, American Society of Hematology/College of American Pathologists; CBF‐AML, core binding factor AML with t(8;21)/AML with inv(16) or t(16;16); MDCA, myelodysplastic cytogenetic abnormalities

Figure 3 summarizes adherence to the American Society of Hematology/College of American Pathologists (ASH/CAP) guidelines [4], which recommend several molecular genetic tests for patients with AML. The guidelines strongly recommend *FLT3‐ITD* testing for all patients with AML; this was performed in 87.9% of the 429 patients who received molecular testing. Recommended testing was also reported for *TET2* (34.0%), *WT1* (29.6%), *IDH1* (46.6%), *IDH2* (46.6%), *DNMT3A* (35.9%), and *TP53* (34.3%). Of the 278 patients without myelodysplastic cytogenetic abnormalities, APL or core binding factor (CBF)‐AML who received molecular testing, testing for *NPM1*, *CEBPA*, and *RUNX1* was reported in 80.9%, 60.1%, and 31.3% of patients, respectively. Of the 22 patients with CBF‐AML who underwent molecular testing, *KIT* was assessed in 18 (81.8%) patients.

### 3.5 Changes in molecular genetic testing over time

**FIGURE 4:**
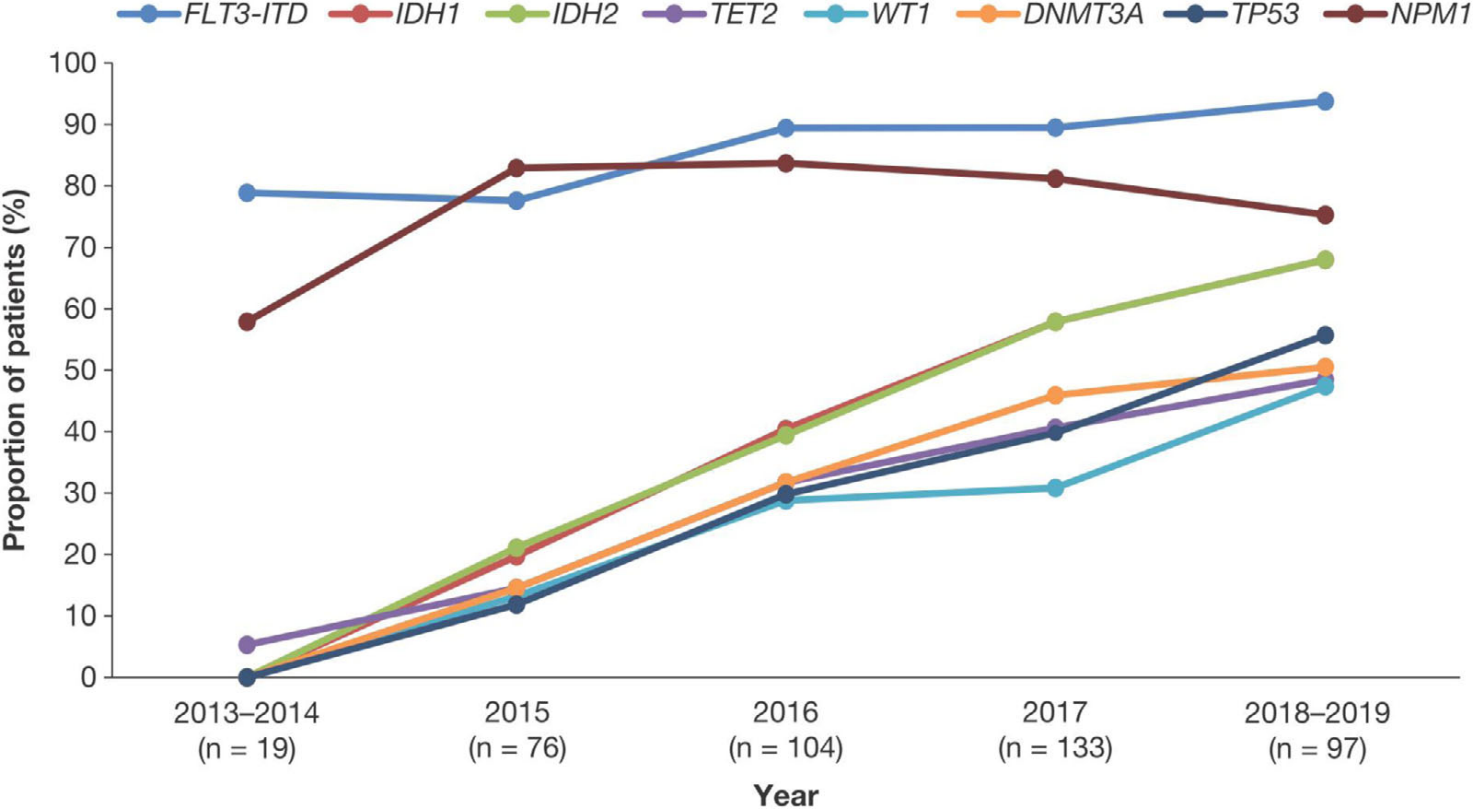
Frequency of testing for specific gene mutations over time. Values represent the proportion of patients tested for gene mutations who were tested for that specific mutation.

To assess if testing patterns have changed since 2013, we compared the mutations tested in patients enrolled early in the Registry (2013‐2016) with those enrolled later (2017‐2019), after the first targeted therapy in AML was approved. These timepoints reflect the publication of updated diagnostic guidelines [4, 5, 32]. In patients enrolled in 2013‐2016, 57.1% of testing used PCR and 35.3% used next‐generation sequencing (NGS), versus 52.6% for PCR and 39.2% for NGS in 2017‐2019. Of the 19 genes analyzed by molecular testing, the mean number of mutations tested for patients enrolled in 2013‐2016 was 6 versus 10 in 2017‐2019 (*P *< .001). Testing rates for individual mutations generally increased between 2013 and 2019 (Figure 4). The largest increases were for *IDH1* and *IDH2* (Figure 4), for which the targeted therapies ivosidenib and enasidenib, respectively, were approved in the United States in 2019 and 2017.

We also assessed how the use of targeted therapies changed over the duration of the Registry to date. Of 24 patients who received targeted therapies (midostaurin, ivosidenib, enasidenib, sorafenib, or gilteritinib) in 2013–2016, 6 (25.0%) received midostaurin and 18 (75.0%) received sorafenib. Given the 2017 US Food and Drug Administration (FDA) approval of enasidenib and the 2018 approval of ivosidenib and gilteritinib, no patients received these drugs before 2017. Of 53 patients who received targeted therapies in 2017‐2019, one (1.9%) received ivosidenib, seven (13.2%) received enasidenib, 20 (37.7%) received midostaurin, 24 (45.3%) received sorafenib, and one (1.9%) received gilteritinib (Table 5). Of 59 patients with mutant *IDH1* or *IDH2*, one (1.7%) received ivosidenib and four (6.8%) received enasidenib. Of 88 patients with either *FLT3‐ITD* or *FLT3‐TKD* mutations, 12 (13.6%) received midostaurin, one (1.4%) received gilteritinib, and 24 (27.2%) received sorafenib. Eighty‐one patients received an investigational drug; 45 enrolled in 2013‐2016, and 36 enrolled in 2017‐2019.

**TABLE 5:** Novel and targeted agents used in the Registry

Agent received, n (%)	Mutation targeted	FDA approval year	2013–2016 (n = 24)	2017–2019 (n = 53)
Ivosidenib	*IDH1*	2018^*^/2019^†^	0 (0.0)	1 (1.9)
Enasidenib	*IDH2*	2017^*^	0 (0.0)	7 (13.2)
Midostaurin	*FLT3*	2017^†^	6 (25.0)	20 (37.7)
Sorafenib	*FLT3‐ITD*	Off‐label	18 (75.0)	24 (45.3)
Gilteritinib	*FLT3*	2018^*^	0 (0.0)	1 (1.9)

FDA, US Food and Drug Administration.

* Approval for patients with R/R‐AML.

† Approval for patients with newly diagnosed AML.

## 4 DISCUSSION

This analysis from the Connect^®^ MDS/AML Disease Registry suggests that, in a clinical practice setting outside of a clinical trial, physicians largely follow testing guidelines, although there is a need for further education to increase testing rates.

In this study, blast assessment was performed manually in 71.7% of Registry patients. The WHO guidelines recommend that blast percentages should be determined manually using 200‐cell leucocyte differential counts of the peripheral blood, and 500‐cell differential counts of all nucleated BM cells in cellular marrow aspirate smears stained with Wright‐Giemsa [32, 34]. A low rate of manual differentials may indicate poor aspirates rather than a lack of awareness of the WHO guidelines. For poor quality aspirates immunohistochemistry may be useful; however, use of flow cytometry to determine blast percentage by counting CD34^+^ cells is discouraged because not all leukemic blasts express CD34, and hemodilution and processing artifacts can cause inaccurate estimation of blast percentage [28, 35]. In a physician survey, respondents reported routinely using flow cytometry, immunohistochemistry, and karyotyping with poor quality aspirates [36].

Molecular testing patterns in AML varied depending on several factors, including patient age, karyotype, treatment setting, and geographic region. Older patients were less likely to undergo molecular testing at community/government sites versus academic sites; similar results were reported in a smaller retrospective study evaluating molecular testing rates in patients with AML [37], where molecular testing was performed in 93% of patients diagnosed at academic centers and 41% of patients diagnosed at outside centers (*P *< .001). This observation may reflect the availability of testing, differences in cooperation between clinical and laboratory experts [38], increased testing as a requirement for clinical trial enrolment at academic centers, or bias that older patients are unlikely to benefit from therapy [39]. It is increasingly acknowledged that age should not be the only factor in making treatment decisions [40], partially because novel, targeted therapies may be better tolerated than conventional therapies. This may lead to an increase in molecular testing rates in older patients.

Multivariable analyses revealed that the only overlapping predictor for molecular testing at both academic and community/government sites was enrolment after 2016. This may suggest changing diagnostic practices as AML testing guidelines were updated [4, 5, 32]. The observation that patients with a normal karyotype were more likely to undergo molecular testing may be due to the perception that molecular genetic testing has a higher yield in normal karyotype patients, dating from an era when guidelines only recommended molecular testing in such patients; now, with the ability to therapeutically target specific mutations, molecular testing has a larger role than simply prognostication. It may also reflect economic factors, reserving costly molecular testing for patients most likely to need accurate risk assessment.

The increase in molecular testing rates for individual genes may be explained by growing awareness of how specific mutations impact survival, and/or by the approval of new targeted therapies. Midostaurin was approved for use in 2017 after being shown to prolong survival for newly diagnosed AML patients with an *FLT3* mutation [11]. Our data show that *FLT3* testing rates have increased since the publication of these data and drug approval. Similarly, testing rates for *IDH2* have increased since enasidenib was approved as a targeted therapy in 2017 [16]. While more patients enrolled in the Registry in 2017‐2019 received targeted therapies versus those enrolled in 2013‐2016, overall molecular testing rates are low when national guideline recommendations are considered. This could be because several drugs, including midostaurin, enasidenib, and ivosidenib, have only recently been approved for first‐line treatment [10, 11, 14]. As the Registry continues to enroll patients, it will be possible to monitor how recently approved agents are introduced into clinical practice.

We noted wide variations in the mutations being tested, revealing the heterogeneity of panels used at different sites. A standardized panel including guideline‐suggested genes may ensure patients are tested for actionable mutations (eg, *FLT3*, *IDH2*) and identify patients eligible for clinical trials and treatment with targeted therapies. The development of a standardized testing panel presents several challenges including: the choice of hotspots/mutations included; variant allele frequency selected; quality and reproducibility of the panel; and financial cost, including insurance reimbursement [23, 40].

The Registry does not capture reasons that may explain the differences in diagnostic approach among treatment sites. Differences in access to testing services, costs of testing, and variations in insurance reimbursements may influence testing patterns. Results may also change as more patients with AML enroll in the Registry. An increase in molecular testing was already seen over a relatively short period of time, and testing frequency may continue to change, particularly as guidelines are updated or new targeted therapies become available.

One important strength of this Registry study is that it represents current routine clinical practice for newly diagnosed patients with AML across practice settings. There were few eligibility criteria other than age (≥55 years) and no comorbidity restrictions. Therefore, the data presented here closely reflect the older patients’ experience.

As with all studies using data from routine clinical practice, this study was subject to limitations concerning lack of randomization and specific protocols for patient assessment and intervention. Despite this, the Registry represents one of the largest prospective cohort studies of patients with AML, treated in geographically diverse and mainly community‐based settings in the United States, and provides insights into their clinical experience. Regardless of the as‐yet‐unknown prognostic value of molecular testing [41, 42], the advent of targeted therapies for AML underscores the importance of molecular characterization of AML in all patients.

## CONFLICT OF INTEREST

DAP: AbbVie, Bristol‐Myers Squibb, Daiichi Sankyo – advisory board member and consultancy; Agios, Forty Seven, Pfizer – advisory board member; Takeda – consultancy; Glycomimetics – data safety and monitoring committee. TIG: Bristol‐Myers Squibb – consultancy; MA: Bristol‐Myers Squibb – advisory board, AbbVie, Bristol‐Myers Squibb, Gilead, Seattle Genetrix, Takeda – speaker panel; CRC, JPM, GGM: no conflicts to disclose; RB: AbbVie, Astex Daiichi Sankyo, Forty Seven, NeoGenomics – consultancy; Bristol‐Myers Squibb – consultancy, honoraria, research funding; Xian‐Janssen – honoraria; KF: Bristol‐Myers Squibb – advisory board member. DLG: AbbVie – consultancy; Alexion – speakers bureau; Astellas, Bristol‐Myers Squibb – advisory board member. RSK: Alexion, Jazz Pharmaceuticals, Novartis – speakers bureau; Agios, Bristol‐Myers Squibb, Daiichi Sankyo, Inc., Incyte, Janssen, Pfizer – consultancy. DAR: Allergan, Amgen, Bristol‐Myers Squibb, Takeda – research funding and consultancy. GJR: AbbVie, Actinum, Agios, Amphivena, Argenx, Astex, Astellas, Bayer, Bristol‐Myers Squibb, Celltrion, Daiichi Sankyo, Eisai, Janssen, Jazz Pharmaceuticals, Novartis, MEI Pharma, Orsenix, Otsuka, Pfizer, Roche/Genentech, Sandoz, Takeda, Trovgene – consultancy, advisory board or data and safety monitoring committee; Cellectis – research funding. MRS: AbbVie – advisory board member, consulting; Boehringer Ingelheim – patents and royalties; Bristol‐Myers Squibb, Selvita – advisory board member; Incyte – advisory board member, research funding; Karyopharm – advisory board member, consultancy, equity ownership; Sunesis – research funding; Takeda, TG Therapeutics – advisory board member, research funding. BLS: Agios – speakers bureau; Alexion, Bristol‐Myers Squibb – advisory board member, consultancy, speakers bureau; Incyte – advisory board member, speakers bureau; Novartis – research funding. MAS: Bristol‐Myers Squibb, Pfizer, Takeda/Millenium – consulting. MAT: Adaptive, Bristol‐Myers Squibb, Doximity, GlaxoSmithKline, Strata Oncology, Syapse Precision Medicine Council, VIA Oncology, UpToDate – consultancy; Doximity – equity; AbbVie, Bristol Myers‐Squibb, CRAB CTC, Denovo, Hoosier Research Network, Lilly, LynxBio, Stata Oncology, Takeda, TG Therapeutics – institutional research funding; SEK: Agios and Bristol‐Myers Squibb – consultancy. CUL, MN, ASS, PK: Bristol‐Myers Squibb ‐ equity and employment. EDF: Bristol‐Myers Squibb – employment. DPS: Astex, Bristol‐Myers Squibb, Onconova, Pfizer, StemLine, Summer Road, Takeda – consultancy. HPE: Agios, Bristol‐Myers Squibb, Jazz Pharmaceuticals, Incyte, Novartis – speakers bureau; AbbVie, Agios, Amgen, Astellas, Bristol‐Myers Squibb, Daiichi Sankyo, Glycomimetics, ImmunoGen, Incyte, Jazz, MacroGenics, Novartis, Pfizer, Seattle Genetics – consultancy; AbbVie, Daiichi Sankyo, ImmunoGen, Macrogenics – research funding; Glycomimetics – data safety and monitoring committee; Covance – independent review committee.

## FUNDING INFORMATION

Celgene Corporation supported the authors in collecting and analyzing the data reported in this Registry. The Connect^®^ MDS/AML Disease Registry is sponsored and funded by Celgene Corporation. The authors received writing and editorial support in the preparation of this manuscript from Lynne Cairns, PhD, of Excerpta Medica, funded by Bristol‐Myers Squibb.

## Supporting information

Supporting Information

## Data Availability

Data requests may be submitted to Celgene, A Bristol Myers Squibb Company at https://vivli.org/ourmember/celgene/ and must include a description of the research proposal.
