# Supplementary material for: Diagnostic and molecular testing patterns in patients with newly diagnosed acute myeloid leukemia in the Connect® MDS/AML Disease Registry
Source: EJHaem. 2020 Jun 30;1(1):58–68. doi: 10.1002/jha2.16 (PMC9176048; doi:10.1002/jha2.16)

**Supplementary Data**

**Diagnostic and molecular testing patterns in patients with newly diagnosed acute myeloid leukaemia in the Connect^®^ MDS/AML Disease Registry**

**Running title:** Diagnostic testing patterns in AML

Daniel A. Pollyea,^1^ Tracy I. George,^2^ Mehrdad Abedi,^3^ Rafael Bejar,^4^ Christopher R. Cogle,^5^ Kathryn Foucar,^6^ Guillermo Garcia-Manero,^7^ David L. Grinblatt,^8^ Rami S. Komrokji,^9^ Jaroslaw P. Maciejewski,^10^ Dennis A. Revicki,^11^ Gail J. Roboz,^12^ Michael R. Savona,^13^ Bart L. Scott,^14^ Mikkael A. Sekeres,^10^ Michael A. Thompson,^15^ Sandra E. Kurtin,^16^ Chrystal U. Louis,^17^ Melissa Nifenecker,^17^ E. Dawn Flick,^18^ Arlene S. Swern,^17^ Pavel Kiselev,^17^ David P. Steensma,^19^ Harry P. Erba^20^

^1^University of Colorado Department of Medicine, Division of Hematology, Aurora, CO, USA; ^2^University of Utah and ARUP Laboratories, Salt Lake City, UT, USA; ^3^University of California, Davis, Sacramento, CA, USA; ^4^University of California San Diego Health, Moores Cancer Center, La Jolla, CA, USA; ^5^University of Florida, Gainesville, FL, USA; ^6^University of New Mexico Health Sciences Center, Albuquerque, NM, USA; ^7^University of Texas MD Anderson Cancer Center, Houston, TX, USA; ^8^NorthShore University HealthSystem, Evanston, IL, USA; ^9^H. Lee Moffitt Cancer Center, Tampa, FL, USA; ^10^Cleveland Clinic Foundation, Cleveland, OH, USA; ^11^Evidera, Bethesda, MD, USA; ^12^Weill Cornell College of Medicine, New York, NY, USA; ^13^Vanderbilt-Ingram Cancer Center, Vanderbilt University School of Medicine, Nashville, TN, USA; ^14^Fred Hutchinson Cancer Research Center, Seattle, WA, USA; ^15^Advocate Aurora Research Institute, Advocate Aurora Health, Milwaukee, WI, USA; ^16^University of Arizona Cancer Center, Tucson, AZ, USA; ^17^Bristol-Myers Squibb, Summit, NJ, USA; ^18^Bristol-Myers Squibb, San Francisco, CA, USA; ^19^Dana-Farber Cancer Institute, Boston, MA, USA; ^20^Duke University, Durham, NC, USA

**Figure S1.** Frequency of testing for 19 genes in 429 patients who had molecular genetic testing.


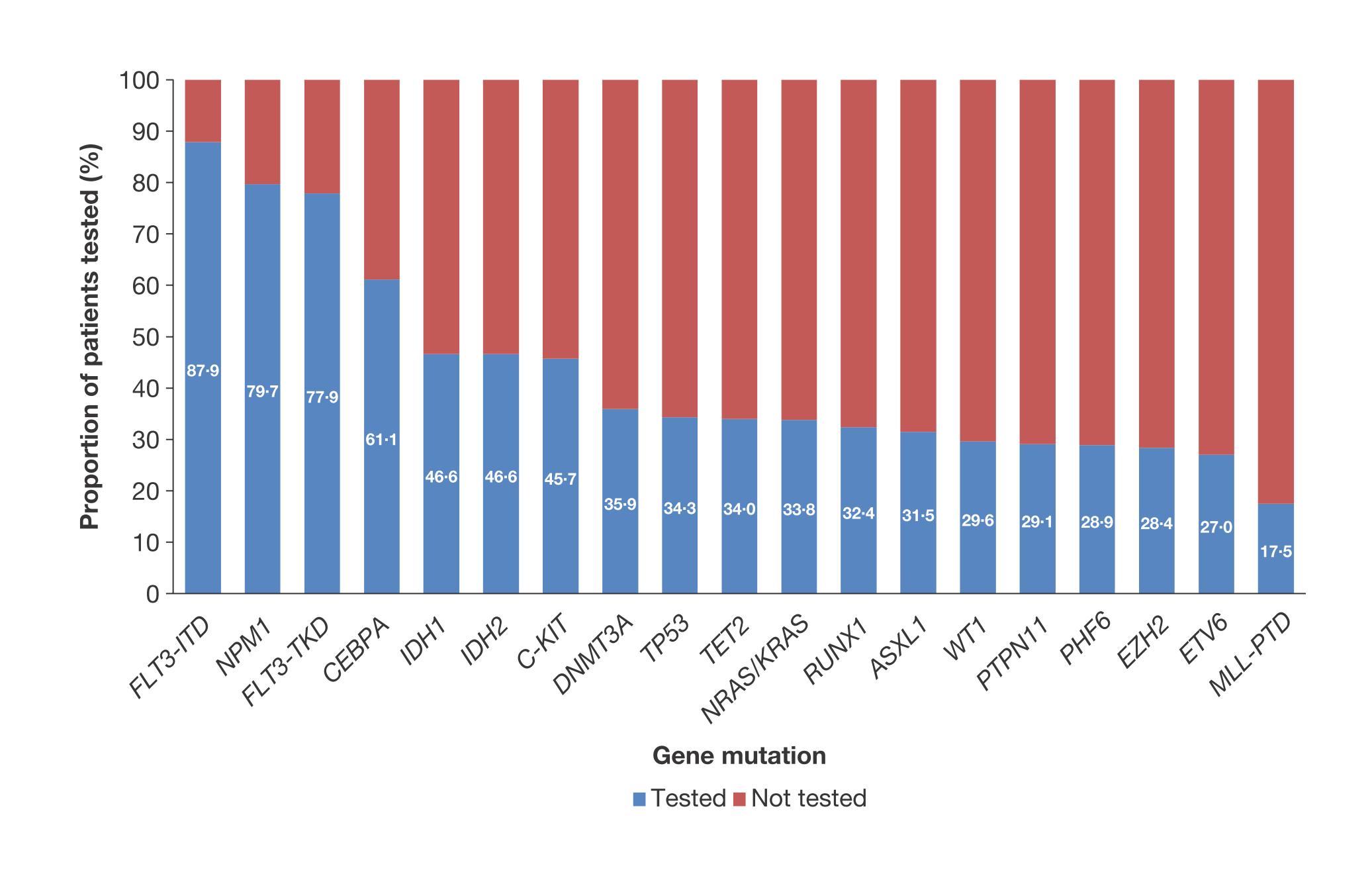

Supplement: Supplementary file 1 — Supporting Information [file JHA2-1-58-s001.docx]
